# Response to the letter from Bushfield and Day: Potential pitfalls of using a correction to normative values in the assessment of acid–base compensation during early ascent to high altitude

**DOI:** 10.1113/EP093358

**Published:** 2025-10-30

**Authors:** Axel Kleinsasser, Benedikt Treml, Sasa Rajsic, Martin Burtscher

**Affiliations:** ^1^ Department of Anaesthesiology & Critical Care Medicine Innsbruck Medical University Innsbruck Austria; ^2^ Department of Sports Sciences University of Innsbruck Innsbruck Austria

**Keywords:** acclimatization, acid–base regulation, altitude

1

We wish to thank Drs Bushfield and Day for their interest in our recent publication on early acclimatization, in which we used this group's metrics for calculating renal reactivity (RR) (Zouboules et al., 2018).

In their letter, Bushfield & Day (2025) propose that normative (here, altitude‐corrected) population level values for base excess may introduce bias and contend that data presentation should be centered on participant level trajectories of $P_{\mathrm{aC}O_{2}}$, actual bicarbonate [HCO_3_^−^(a)] and arterial pH (pH_a_). They raise two main concerns:

- That the derivation of base excess used by us was not developed to assess early acclimatization responses.
- The use of assumed normative values was potentially misleading, particularly when researchers have each participant's actual measured values available.

For the base excess issue (a), we would like to note that Zubieta‐Calleja and coworkers state that in acclimatized residents, a hypocapnic $P_{\mathrm{aC}O_{2}}$ at the altitude of La Paz, Bolivia, (3500 m) does not exist, rather a normocapnic $P_{\mathrm{aC}O_{2}}$ for a corrected altitude (Zubieta‐Calleja et al., 2011). To observe how fast base excess reacts and then approaches zero in our experiment we used their altitude‐corrected formula. We should have stated in context in the Methods section that we calculated base excess in this manner also for this purpose. Bushfield and Day rightly criticize this deficit, since we failed to mention this.

Regarding the issue that the normative values were potentially misleading (b), we would like to point out that directly measured values are on display in Table 1 ($P_{\mathrm{aC}O_{2}}$) and Figure 2 (Skalla et al., 2026): in Figure 2 measured and calculated values are given in four panels. In this way, HCO_3_^−^(a), pH_a_, as well as calculated renal reactivity and base excess are presented in parallel. We feel that we have committed neither any ‘normalizing away’ nor ‘obscuring’ of direction and magnitude of any response. The presentation, however, might have been clearer.

We would like to amend a note on HCO_3_^−^(a) and acclimatization: HCO_3_^−^(a) is a variable derived from Henderson–Hasselbalch equation using pH_a_ and $P_{\mathrm{aC}O_{2}}$, such as:

$$\mathrm{HC}{O_{3}}^{-}\left(a\right)=0.0304\times P_{\mathrm{aC}O_{2}}\times 10^{\left(\mathrm{pH}-6.095\right)}$$

The search for acute‐exposure (<1 week) and acclimatized values of HCO_3_^−^(a) and $P_{\mathrm{aC}O_{2}}$ guides us to the excellent review of Ramirez‐Sandoval et al. (2016). Here, calculated and some measured HCO_3_^−^(a) and $P_{\mathrm{aC}O_{2}}$ values are reported with some limitations; however, the altitude of our experiment (3100 m) should result in a derived HCO_3_^−^(a) of 21.7 mEq/L (acute exposure, <1 week) and 18.3 mEq/L in acclimatized individuals. One notable limitation is the assumption of a pH_a_ of 7.4 in most calculations (Ramirez‐Sandoval et al., 2016). Considering available data, it seems reasonable to assume acclimatization to 3100 m when HCO_3_^−^(a) reaches the approximate threshold of 18.3 mEq/L and, similarly, altitude‐corrected base excess reaches zero. Based on the contribution of Bushfield and Day we now regret that we failed to present this information in the original submission. Similar to the approach of base excess converging toward zero, one could describe the trajectory of calculated HCO_3_^−^(a) from lowland values of 24.5 mEq/L to the acclimatized value at an altitude of 21.7 (acute exposure) and 21.3 mEq/L (chronic exposure) as described in the work of Ramirez‐Sandoval et al. (2016).

The lack of direct measurements commands further studies of HCO_3_^−^(a), pH_a_ and $P_{\mathrm{aC}O_{2}}$ after acute or chronic exposure to altitude in healthy individuals. Missing correction for altitude may also have medical implications: Negrete‐Pedraza and coworkers reported that in Mexico City (2250 m) children were falsely assumed to suffer from renal tubular acidosis on the basis of a defect of the chloride–HCO_3_^−^ anion exchanger in the renal tubules (Negrete‐Pedraza et al., 2024). The children showed HCO_3_^−^ values below normal (i.e. below lowland values) and many were thought to suffer from an acid–base disorder such as renal tubular acidosis.

Presentation of normalized values can be problematic, we agree on that, and we admit we could have done better in presentation such as always stating what a particular calculated parameter means, specifically in a way that a less seasoned reader would not risk conflating direct measurement with normalized calculation. In the case of our work, bare observation of HCO_3_^−^(a) might replace base excess or renal reactivity calculations as $P_{\mathrm{aC}O_{2}}$ was constant due to staging in our observation. A useful addition for judging *renal reactivity* could be reporting how and to what extent chloride compensates renal HCO_3_^−^ excretion and how this is depicted in the anion gap:

$$\mathrm{Anion}\mathrm{gap}\left(\mathrm{mEq}/L\right)=\left(\mathrm{Sodium}+\mathrm{Potassium}\right)-\left(\mathrm{Chloride}+\mathrm{HC}{O_{3}}^{-}\right)$$

This may be of interest since salt retention might have implications with oedema formation. There is work left to do.

In conclusion, we would like to thank Drs Bushfield and Day for their valuable comments and the editor for the opportunity to clarify and to add information to our work.

## AUTHOR CONTRIBUTIONS

All authors have read and approved the final version of this manuscript and agree to be accountable for all aspects of the work in ensuring that questions related to the accuracy or integrity of any part of the work are appropriately investigated and resolved. All persons designated as authors qualify for authorship, and all those who qualify for authorship are listed.

## CONFLICT OF INTEREST

None declared.

## FUNDING INFORMATION

None.
